# Culture Temperature Affects Human Chondrocyte Messenger RNA Expression in Monolayer and Pellet Culture Systems

**DOI:** 10.1371/journal.pone.0128082

**Published:** 2015-05-26

**Authors:** Akira Ito, Momoko Nagai, Junichi Tajino, Shoki Yamaguchi, Hirotaka Iijima, Xiangkai Zhang, Tomoki Aoyama, Hiroshi Kuroki

**Affiliations:** 1 Department of Motor Function Analysis, Human Health Sciences, Graduate School of Medicine, Kyoto University, Kyoto, Japan; 2 Japan Society for the Promotion of Science, Tokyo, Japan; 3 Department of Development and Rehabilitation of Motor Function, Human Health Sciences, Graduate School of Medicine, Kyoto University, Kyoto, Japan; University of Massachusetts Medical, UNITED STATES

## Abstract

Cell-based therapy has been explored for articular cartilage regeneration. Autologous chondrocyte implantation is a promising cell-based technique for repairing articular cartilage defects. However, there are several issues such as chondrocyte de-differentiation. While numerous studies have been designed to overcome some of these issues, only a few have focused on the thermal environment that can affect chondrocyte metabolism and phenotype. In this study, the effects of different culture temperatures on human chondrocyte metabolism- and phenotype-related gene expression were investigated in 2D and 3D environments. Human chondrocytes were cultured in a monolayer or in a pellet culture system at three different culture temperatures (32°C, 37°C, and 41°C) for 3 days. The results showed that the total RNA level, normalized to the threshold cycle value of internal reference genes, was higher at lower temperatures in both culture systems. Glyceraldehyde-3-phosphate dehydrogenase (*GAPDH*) and citrate synthase (*CS*), which are involved in glycolysis and the citric acid cycle, respectively, were expressed at similar levels at 32°C and 37°C in pellet cultures, but the levels were significantly lower at 41°C. Expression of the chondrogenic markers, collagen type IIA1 (*COL2A1*) and aggrecan (*ACAN*), was higher at 37°C than at 32°C and 41°C in both culture systems. However, this phenomenon did not coincide with SRY (sex-determining region Y)-box 9 (*SOX9*), which is a fundamental transcription factor for chondrogenesis, indicating that a *SOX9*-independent pathway might be involved in this phenomenon. In conclusion, the expression of chondrocyte metabolism-related genes at 32°C was maintained or enhanced compared to that at 37°C. However, chondrogenesis-related genes were further induced at 37°C in both culture systems. Therefore, manipulating the culture temperature may be an advantageous approach for regulating human chondrocyte metabolic activity and chondrogenesis.

## Introduction

Hyaline articular cartilage covers the epiphyseal bone end and plays a role as a low-friction, wear-resistant, and load-bearing material within a synovial joint. Cartilage is composed of water, extracellular matrix (ECM), which is mainly composed of type II collagen and aggrecan, and cartilage-specific cells called chondrocytes [1]. While chondrocytes play a vital role in ECM anabolism and catabolism, articular cartilage displays a limited capacity for self-repair, due to hypocellularity and hypovascularity [2,3]. Therefore, tissue engineering and cell-based therapy have been explored for articular cartilage regeneration [4].

Autologous chondrocyte implantation (ACI) is a promising cell-based technique for repairing articular cartilage defects [5]. However, there are several issues to overcome in order to achieve plenary regeneration by ACI [4]. Chondrocyte de-differentiation is one of the issues. For ACI, chondrocytes need to be isolated from a minor load-bearing area of the knee and cell expansion is required to obtain a sufficient amount of cells. When chondrocytes are isolated from the ECM and cultured in monolayer conditions, the chondrogenic phenotype disappears during de-differentiation and a fibroblastic phenotype appears [6,7]. De-differentiation occurs immediately in monolayer conditions [8,9]. Synthesis of type II collagen and aggrecan, which are specific markers of articular chondrocytes, decreases and type I collagen synthesis increases [6,7]. Hyaline cartilage ECM cannot be produced by these de-differentiated chondrocytes, and instead, fibrocartilage-like ECM is formed [10]. The fibro-cartilage-like ECM cannot endure mechanical and chemical stresses affecting an articular joint, which, ultimately, leads to degeneration [11]. Therefore, preventing chondrocyte de-differentiation and promoting re-differentiation are crucial for the regeneration of the hyaline cartilage and are expected to improve clinical outcomes.

To maintain the articular chondrocyte-specific phenotype and to re-differentiate de-differentiated chondrocytes, numerous investigations have been conducted. These studies revealed that microenvironment elements such as matrix scaffolds [12–14], growth factors [15–17], mechanical stimuli [18–20], osmolality [21], and oxygen pressure [22–24] affect chondrocyte metabolism and phenotype. In general, an *in vivo* mimicking microenvironment such as a three-dimensional (3D) or a hypoxic microenvironment allows the maintenance of the chondrocyte phenotype and the ECM synthesis promotion [25]. However, only few studies focused on the thermal environment [26–28], which is an important parameter for all cell types. The temperature within a human knee joint is approximately 32°C, which is 4–5°C lower than the inner body temperature [29,30]. Thus, chondrocytes may retain their phenotype when cultured at an *in vivo* mimicking temperature.

Recently, we investigated the effects of culture temperatures on chondrocyte metabolism using immature porcine chondrocytes [31,32]. We showed that temperature affects chondrocyte proliferation, specific gene expression, and ECM synthesis, and that a culture temperature of 37°C would be an appropriate temperature to induce chondrogenesis. However, it also suggested the possibility that differences in culture systems (two-dimensional [2D] or 3D) could alter the effects of culture temperature. In addition, differences based on cell species and cell maturity could also exist. Thus, to improve existing knowledge, the effects of culture temperature on mature human chondrocytes in 2D and 3D environments must be investigated. The purpose of this study was to elucidate the effects of different culture temperatures on human chondrocyte metabolism- and phenotype-related gene expression in 2D and 3D environments.

## Materials and Methods

### Ethical statement

The Ethics Committee of the Faculty of Medicine at Kyoto University approved the procedure (approval no. 944), and written informed consent was obtained from the donor.

### Chondrocyte isolation

**Fig 1:**
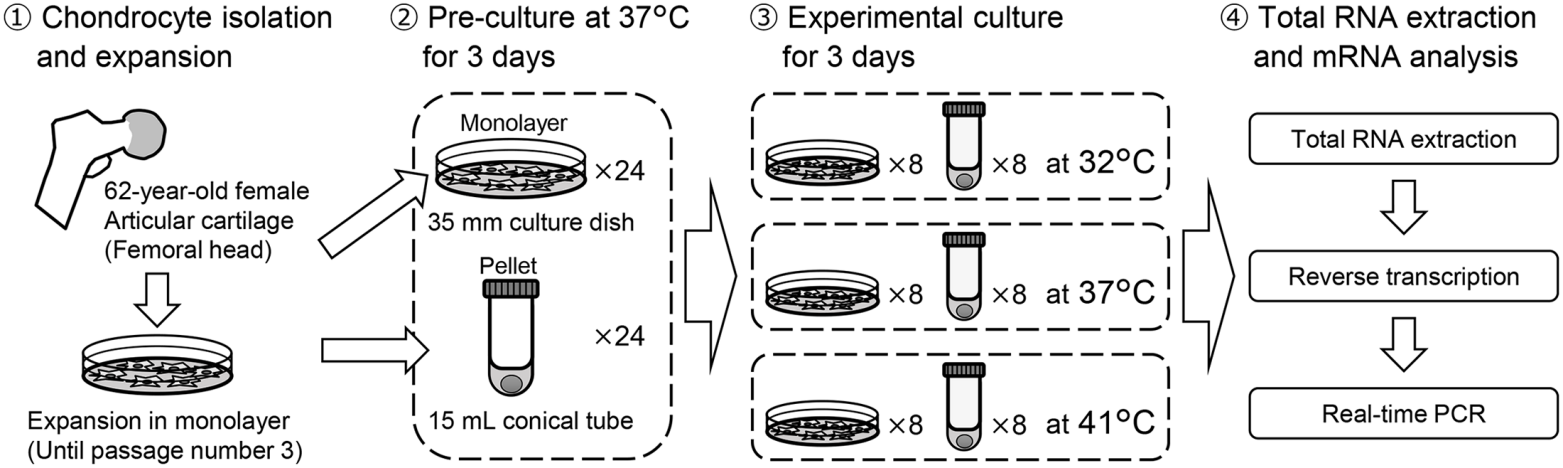
Experimental design.

The experimental design is described in Fig 1. Human articular cartilage (International Cartilage Repair Society grade 0) was obtained from the femoral head of a 62-year-old woman. It was extracted while performing a bipolar hip arthroplasty. Chondrocytes were aseptically isolated as previously described [33]. The isolated cells were resuspended in culture medium (Dulbecco’s modified Eagle medium/Ham’s F12 [DMEM/Ham’s F12; Nacalai Tesque Inc., Kyoto, Japan] containing 10% fetal bovine serum [FBS; Hyclone, Logan, UT, USA], 50 U/mL penicillin [Nacalai Tesque Inc.], and 50 μg/mL streptomycin [Nacalai Tesque Inc.]) and were seeded in a 100-mm-diameter culture dish. Chondrocytes were expanded in a CO_2_ incubator (5% CO_2_, 37°C, and 95% humidity) until the third passage to obtain an adequate quantity of cells.

### Monolayer and pellet culture at three different temperatures

For monolayer culture, the expanded chondrocytes were trypsinized and sub-cultured at 1 × 10^4^ cells/cm^2^ in culture medium into 24 culture dishes (35 mm diameter). For pellet cultures, chondrocytes were trypsinized, washed with culture medium, and resuspended in chondrogenic medium (Lonza, Walkersville, MD, USA: chondrogenic basal medium, plus ITS + supplement, ascorbate, dexamethasone, l-glutamine, sodium pyruvate, proline, and GA-1000) supplemented with 10 ng/mL of recombinant human transforming growth factor-beta 3 (R&D Systems Inc., Minneapolis, MN, USA). Aliquots of 2.5 × 10^5^ cells in 500 μL of the chondrogenic medium in 24 15-mL-polypropylene conical tubes were centrifuged at 250 × *g* for 5 min to form a cell pellet. The monolayer culture cells and the pelleted cells were pre-cultured at 37°C for 3 days. After pre-culture, the dishes and the tubes were exposed to three distinct temperatures for 3 additional days by transferring them into three distinct CO_2_ incubators set at 32°C, 37°C, and 41°C (8 dishes and tubes for each group). These culture temperatures were defined as follows: 32°C, the physiological intra-articular temperature [29,30]; 37°C, the inner body temperature, which is conventionally used; and 41°C, high temperature, the threshold temperature for mammalian cell survival [34,35].

### Total RNA extraction and real-time PCR

After 3 days of culture, chondrocytes were harvested to analyze gene expression. Total RNA was extracted using the RNeasy Mini Kit following the manufacturer’s protocol (Qiagen Inc., Valencia, CA, USA) and purified by RNase-free DNase on-column incubation. The amount of the extracted total RNA was estimated by measuring the absorbance at 260 nm (A_260_). An A_260_ reading of 1.0 is corresponds to 40 μg/mL of RNA [36]. In addition, its purity was confirmed by calculating the A_260_/A_280_ ratio (>2.0) for all samples using a NanoDrop 2000 spectrophotometer (Thermo Fisher Scientific, Wilmington, DE, USA).

Reverse transcription was performed using the ReverTra Ace qPCR RT Kit (Toyobo, Osaka, Japan), according to the manufacturer’s protocol. Total RNA (350 ng) was reverse-transcribed for 15 min at 37°C to synthesize cDNA, followed by incubation at 98°C for 5 min to deactivate the enzyme. Real-time PCR was performed using the Applied Biosystems7500 Real-Time PCR System (Life Technologies Corporation, Carlsbad, CA, USA). cDNA templates corresponding to 4.375 ng of total RNA were amplified using Power SYBR Green PCR Master Mix (Life Technologies Corporation) in 25 μL reaction containing 1× Power SYBR Green PCR Master Mix, 0.2 μM of each gene-specific primer, and deionized water. The mixture was initially heated at 95°C for 10 min, followed by 40 cycles of denaturation at 95°C for 15 s and annealing and extension at 60°C for 60 s. Following amplification, melting curves were obtained to ensure that a primer-dimers or non-specific products were eliminated or minimized. The following target genes were examined: glyceraldehyde-3-phosphate dehydrogenase (*GAPDH*), citrate synthase (*CS*), beta-actin (*ACTB*), collagen type IIA1 (*COL2A1*), collagen type IA1 (*COL1A1*), aggrecan (*ACAN*), and SRY (sex-determining region Y)-box 9 (*SOX9*). Ribosomal protein L13a (*RPL13a*) and tyrosine 3-monooxygenase/tryptophan 5-monooxygenase activation protein, zeta (*YWHAZ*) were used as internal reference genes. Primer sequences are provided in Table 1.

**Table 1:** Primer sequences for real-time PCR.

Symbol	Gene name	Accession number	Primer sequence (5′-3′)	Amplicon size (bp)
GAPDH	Glyceraldehyde-3-phosphate dehydrogenase	NM_002046	F-TCTCCTCTGACTTCAACAGCGAC	126
			R-CCCTGTTGCTGTAGCCAAATTC	
CS	Citrate synthase	NM_004077.2	F-TCTGGAACACACTCAACTCAGG	150
			R-TGTACAGCTGAGCAACCAAC	
ACTB	Beta-actin	NM_001101.2	F-GCCCTGAGGCACTCTTCCA	100
			R-CGGATGTCCACGTCACACTTC	
COL2A1	Collagen, type II, alpha 1	NM_001844	F-GGAATTCGGTGTGGACATAGG	92
			R-ACTTGGGTCCTTTGGGTTTG	
COL1A1	Collagen, type I, alpha 1	NM_000088	F-CAGAACGGCCTCAGGTACCA	83
			R-CAGATCACGTCATCGCACAAC	
ACAN	Aggrecan	NM_001135	F-GAATGGGAACCAGCCTATACC	98
			R-TCTGTACTTTCCTCTGTTGCTG	
SOX9	SRY (sex-determining region Y)-box 9	NM_000346	F-AGCGAACGCACATCAAGAC	110
			R-GCTGTAGTGTGGGAGGTTGAA	
RPL13a	Ribosomal protein L13a	NM_012423	F-AAGTACCAGGCAGTGACAG	100
			R-CCTGTTTCCGTAGCCTCATG	
YWHAZ	Tyrosine 3-monooxygenase/tryptophan 5-monooxygenase activation protein, zeta	NM_003406.3	F-TGCTTGCATCCCACAGACTA	126
			R-AGGCAGACAATGACAGACCA	

The data obtained by real-time PCR were analyzed by the comparative threshold cycle method. Briefly, the target gene quantity was normalized to that of *RPL13a* and *YWHAZ*, which are known to be stable under different thermal environments [37]. The mean value of the calibration sample (the cells cultured in monolayer at 32°C) was set to 1 and the values obtained for each of the other conditions were calculated relative to that of the calibration sample. Before using the comparative threshold cycle method for quantitation, we performed a validation experiment and the absolute value of the slope of log input amount versus delta threshold cycle was less than 0.1. To normalize the amount of total RNA, total RNA in each sample was divided by the corresponding threshold cycles of the internal reference genes to compensate for the difference in cell numbers.

### Statistical analysis

The software JMP 11 (SAS Institute, Cary, NC, USA) was used for statistical analysis. The values are reported as means ± standard deviation (SD) for normalized total RNA quantification and as median and interquartile range for the mRNA expression. Statistical significance was determined using one-way analysis of variance with the Tukey-Kramer post-hoc multiple comparison test for normalized total RNA quantification and using the Steel-Dwass test for mRNA expression analysis. Wilcoxon rank sum test was used for comparison between monolayer and pellet culture systems. In all cases, *P* < 0.05 was considered significant.

## Results

### Normalized total RNA quantification

**Fig 2:**
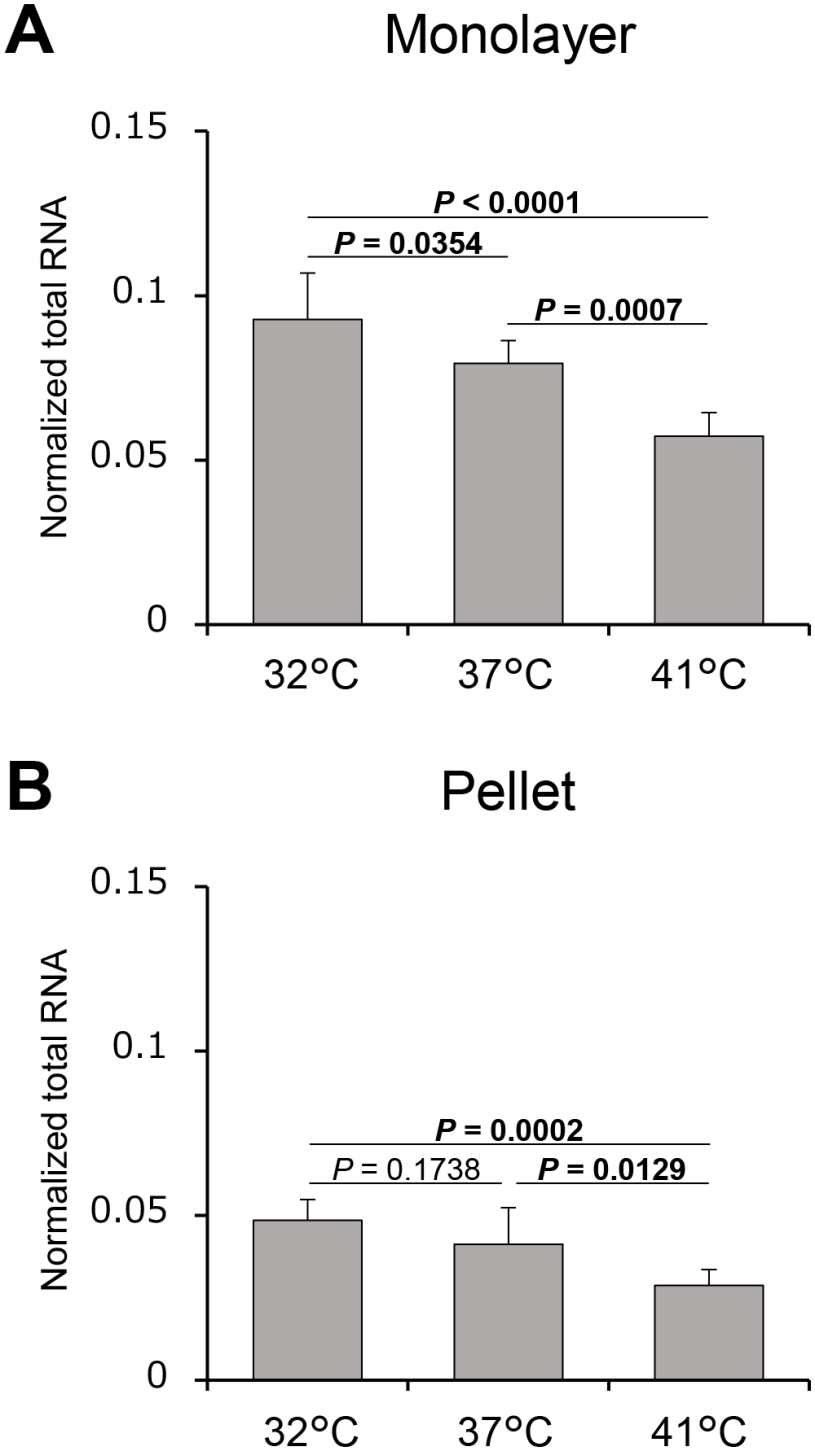
Normalized total RNA. Normalized total RNA at three different culture temperatures was estimated in monolayer (A) and pellet (B) culture systems. Values are expressed as the mean ± standard deviation. *n* = 8 dishes or pellets/group. Significantly different values (*P* < 0.05) are written in bold.

Total RNA normalized to the threshold cycles of the internal reference genes decreased in a temperature-dependent manner. No significant difference was observed at 32°C and 37°C in pellet cultures (Fig 2B; *P* = 0.1738).

### mRNA expression analysis

**Fig 3:**
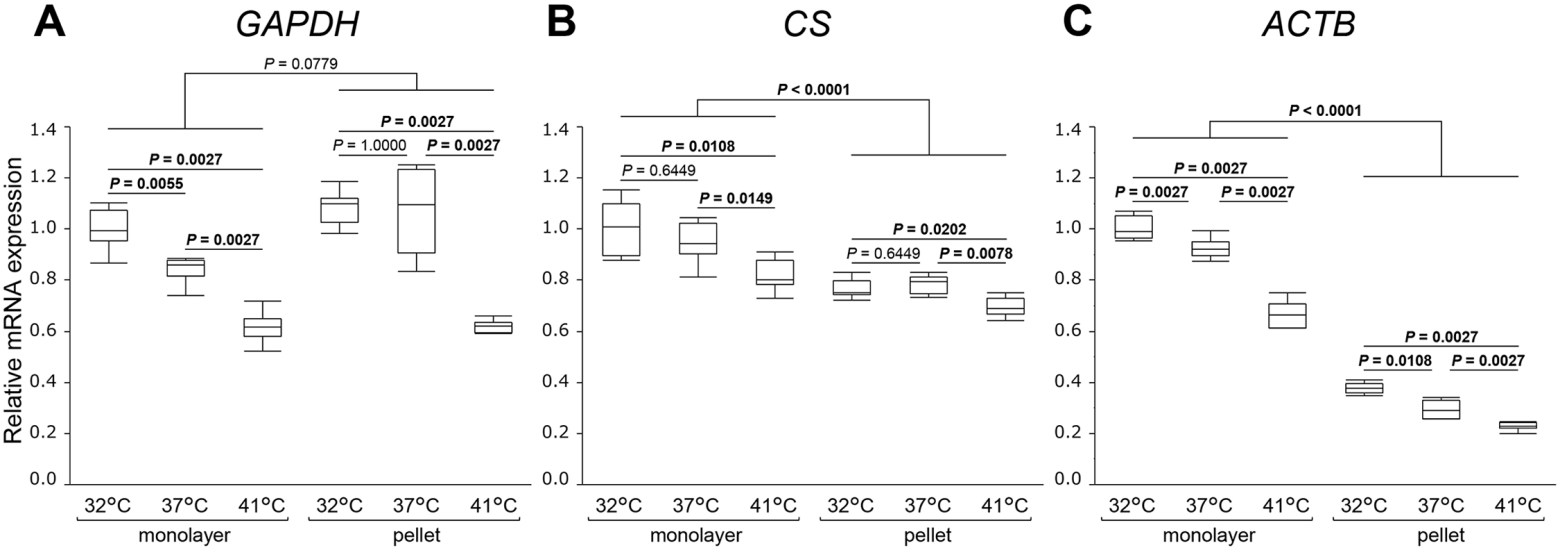
Chondrocyte metabolism-related gene expression. *GAPDH* (A), *CS* (B), and *ACTB* (C) relative mRNA expression was measured at three different culture temperatures in monolayer and pellet culture systems. Values are expressed as median and interquartile range. *n* = 8 dishes or pellets/group. Significantly different values (*P* < 0.05) are written in bold.

To elucidate the effects of temperature on chondrocyte metabolism in monolayer and pellet cultures, *GAPDH*, *CS*, and *ACTB* mRNA expression was evaluated. *GAPDH* was downregulated in a temperature-dependent manner in monolayer cultures (Fig 3A). In pellet cultures at 32°C and 37°C, *GAPDH* mRNA expression was comparable to that of the monolayer culture at 32°C, but *GAPDH* mRNA expression was significantly lower at 41°C than that at 32°C and 37°C (*P* = 0.0027 in both cases). In both culture systems, *CS* mRNA expression was significantly lower at 41°C when compared to that at 32°C (*P* = 0.0108 in monolayer; *P* = 0.0202 in pellet) and 37°C (*P* = 0.0149 in monolayer and *P* = 0.0078 in pellet) (Fig 3B). Similar to *GAPDH*, *ACTB* mRNA expression was downregulated in a temperature-dependent manner (Fig 3C). When comparing monolayer and pellet cultures, *CS* and *ACTB* mRNA expression was significantly lower in pellet cultures than in monolayer cultures (*CS*: *P* < 0.0001; *ACTB*: *P* < 0.0001), while *GAPDH* mRNA expression was not different (*P* = 0.0779) (Fig 3A, 3B, and 3C).

**Fig 4:**
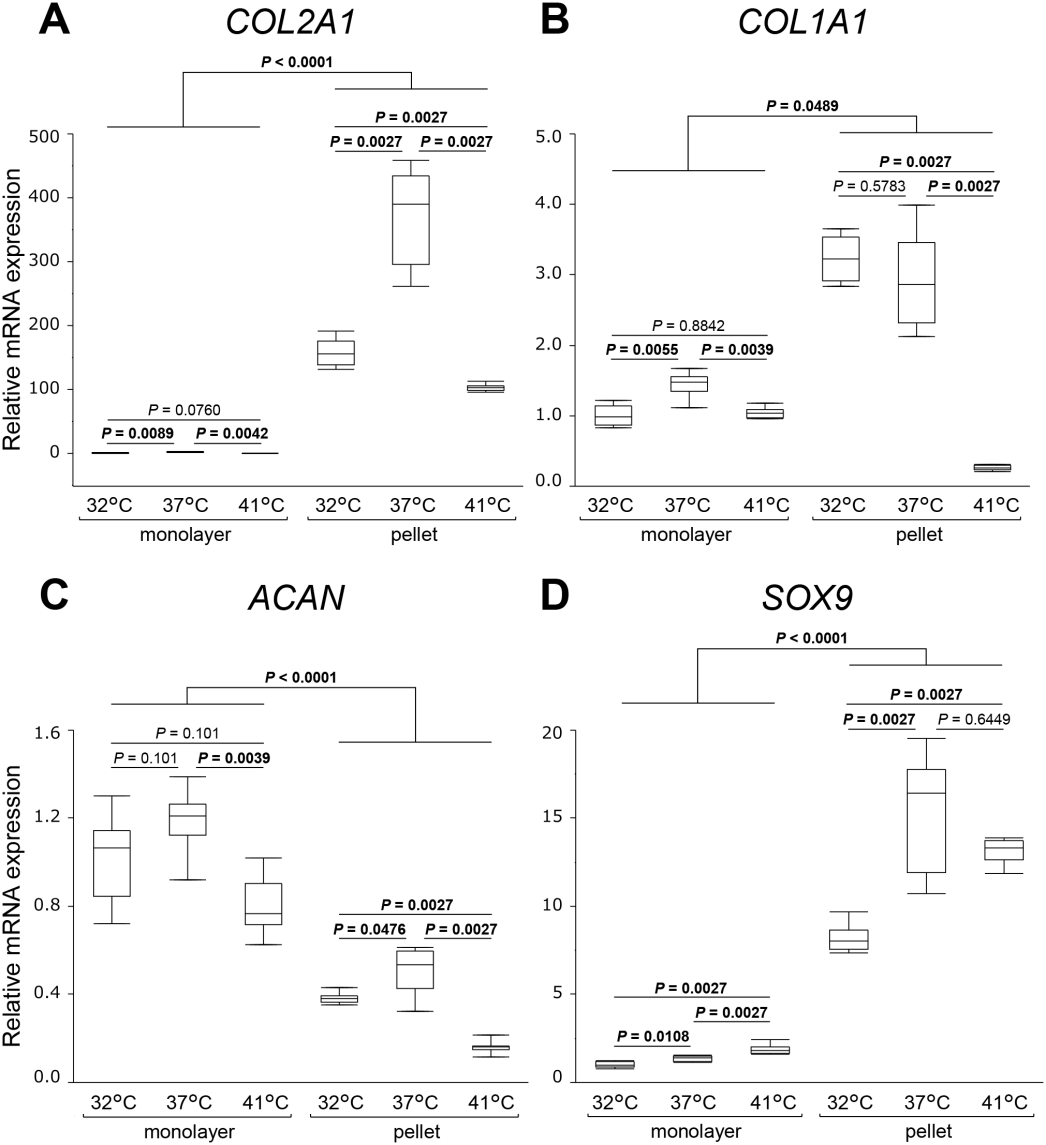
Chondrocyte phenotype and extracellular matrix-related gene expression. *COL2A1* (A), *COL1A1* (B), *ACAN* (C), and *SOX9* (D) relative mRNA expression was measured at three different culture temperatures in monolayer and pellet culture systems. Values are expressed as median and interquartile range. *n* = 8 dishes or pellets/group. Significantly different values (*P* < 0.05) are written in bold.

To clarify the effects of temperature on the chondrocyte phenotype and ECM-related genes, *COL2A1*, *COL1A1*, *ACAN*, and *SOX9* mRNA expression was investigated. In monolayer cultures, *COL2A1* was significantly higher at 37°C compared to that of the monolayer culture at 32°C (2.2-fold, *P* = 0.0089) and 41°C (3.1-fold, *P* = 0.0042) (Fig 4A). In pellet cultures, *COL2A1* expression was significantly higher than that in the monolayer (*P* < 0.0001), retaining the effectiveness of the culture temperature of 37°C. In monolayer cultures, *COL1A1* expression was also slightly, but significantly, higher at 37°C compared to that at 32°C (1.4-fold, *P* = 0.0055) and 41°C (1.4-fold, *P* = 0.0039) (Fig 4B). In pellet cultures, *COL1A1* expression was higher than in monolayer cultures (*P* = 0.0489). However, the difference between monolayer and pellet cultures for *COL1A1* expression was considerably small compared to that for *COL2A1*. There was no significant difference between 32°C and 37°C in pellet cultures (*P* = 0.5783). *ACAN* showed a distinct trend when compared to other genes (Fig 4C). In pellet cultures, *ACAN* expression was significantly lower than that in monolayer cultures (*P* < 0.0001), while its expression was higher at 37°C when compared to that at 32°C and 41°C, especially in pellet cultures (*P* = 0.0476 and 0.0027, respectively). *SOX9* was significantly enhanced at higher temperature (1.4-fold at 37°C, *P* = 0.0108 and 1.9-fold at 41°C, *P* = 0.0027 compared with that at 32°C, respectively) in monolayer cultures (Fig 4D). *SOX9* expression in pellet cultures was strongly enhanced in comparison with that in monolayer cultures (*P* < 0.0001), and its expression at 37°C and 41°C was higher than that at 32°C (*P* = 0.0027 for both).

## Discussion

In the current study, we elucidated the effects of different culture temperatures on chondrocyte metabolism- and phenotype-related gene expression in monolayer and pellet culture systems. To the best of our knowledge, this is the first report investigating differences in human chondrocyte mRNA expression at different culture temperatures in 2D and 3D environments. Here, we provide evidence suggesting that the expression of cellular respiration-related genes is maintained or enhanced at 32°C compared to that at 37°C and 41°C. However, the expression of chondrogenesis-related genes was further induced at 37°C than at 32°C and 41°C in both monolayer and pellet culture systems.

### Effects of culture temperature and culture system on chondrocyte metabolism

In general, cells are cultured at 37°C *in vitro* to mimic the inner body temperature from which they originate. However, chondrocytes within the knee joint are chronically exposed to a lower temperature of approximately 32°C [29,30]. It is well-known that lowering culture temperature induces an immediate reduction in growth rate and metabolism such as reduction of glucose consumption, lactate production, and oxygen uptake [38,39]. The growth rate reduction is caused by a cell cycle arrest in the G_0_/G_1_ phase [40]. On the other hand, low culture temperature is also known to enhance the specific productivity of Chinese hamster ovary cells expressing erythropoietin, and glucose consumption and lactate production rates were elevated at 30°C [41]. Thus, these effects are dependent on cell types and target proteins. However, only few studies focused on chondrocytes cultured at 32°C, *in vitro*, to mimic the intra-articular temperature from which they originate. Only one study, reported by Kocaoglu *et al*. [27], showed that, in porcine osteochondral explants, the mRNA expression of the metabolism-related genes, *ACTB*, *GAPDH*, and hypoxia-inducible transcription factor 1 alpha, was higher when the explants were cultured at 32°C than at other temperatures. In the current study, we performed total RNA quantification in order to estimate human chondrocytes metabolic activity. Our results showed that normalized total RNA was higher at lower temperatures in both monolayer and pellet culture systems (Fig 2A and 2B). These results are in agreement with the study by Kocaoglu *et al*. [27] and indicate that the culture temperature of 32°C is probably suitable for human chondrocyte metabolism. The difference in normalized total RNA between the monolayer and pellet culture system was thought to be attributed to the amount of polysaccharides, such as proteoglycan, which influences the efficacy of total RNA extraction [42]. Thus, a direct comparison would be inadequate.

To further understand the influence of temperature on metabolism, cellular respiration levels were estimated by analyzing the expression of the glycolysis and citric acid cycle (Krebs cycle)-related genes *GAPDH* and *CS*, respectively. *GAPDH* encodes a member of the glyceraldehyde-3-phosphate dehydrogenase protein family. This enzyme is mainly involved in glucose breakdown by catalyzing the sixth step of glycolysis. However, it is also involved in a number of diverse non-glycolytic cellular processes such as apoptosis, neuronal disorders, viral pathogenesis, and endocytosis [43]. The enzyme encoded by *CS* catalyzes the condensation of oxaloacetate and acetyl coenzyme A to form citrate in the initial step of the citric acid cycle. It has been extensively used as a metabolic marker in assessing oxidative and respiratory capacity [44]. Our results showed that *GAPDH* was higher at lower temperatures in monolayer cultures. Similar levels were observed at 32°C and 37°C in pellet cultures, while it was significantly repressed at 41°C (Fig 3A). *CS* levels were similar at 32°C and 37°C in both culture systems, while it was significantly repressed at 41°C (Fig 3B). Thus, physiological temperatures (32–37°C) may not influence *GAPDH and CS* expression in pellet culture, but a high temperature (41°C) may induce adverse effects on cellular respiration. When comparing culture systems, *CS* was lower in pellet than in monolayer cultures. This result could be attributed to oxygen tension, which can reach zero in the central region in pellet cultures [45]. Taken together, our results indicate that cellular respiration-related genes were active even if chondrocytes were cultured at a low temperature (32°C), corresponding to the temperature of the human knee joint from which chondrocytes originate.

*ACTB* encodes one of the six different actin proteins, which are components of the cytoskeletal microfilaments. The actin microfilaments are involved in many fundamental cellular events, including alteration of cell shape, movement of organelles, cell migration, and ECM assembly [46]. Our results showed that *ACTB* was lower at higher temperatures in both culture systems. It has been reported that de-differentiation is mediated by actin polymerization and stress fiber formation [47,48]. However, the involvement of actin microfilaments in de-differentiation remains controversial. Idowu *et al*. [49] revealed that actin microfilaments within chondrocytes in agarose were polymerized and organized into a complete cortical network. This actin microfilament organization was similar to that of the *in situ* chondrocytes within the cartilage. Therefore, they do not support the hypothesis that phenotypic modulation, which occurs when chondrocytes are cultured in monolayers, is mediated by actin polymerization *per se*. We speculate that the formation of actin microfilament bundles such as stress fibers, not the actin microfilament cortical network, may be involve in the de-differentiation. Thus, an important determinant of the phenotype maybe the distributional property of actin microfilaments rather than its amount. *ACTB* expression level did not appear to be correlated with chondrogenesis-related gene expression levels, demonstrating, at least in part, our speculation. Our results also showed that higher *ACTB* levels were expressed at 32°C in both culture systems, which is consistent with Kocaoglu *et al*. results [27]. This trend was similar to that of normalized total RNA. This tendency is most likely related to the fact that actin is one of the most abundant proteins in eukaryotes. When comparing the two culture systems, *ACTB* was lower in pellet than in monolayer cultures. Application of prolonged mechanical compression such as static compression results in the loss of the uniform cortical distribution of the actin microfilament networks [50]. In pellet cultures, cells were centrifuged and cultured in a high-density environment. Those external mechanical stimuli are thought to downregulate *ACTB* expression in the pellet culture system. *ACTB* and *GAPDH* are frequently used for PCR analyses as internal reference control genes. Indeed, the expression of these genes is not altered between 33°C and 37°C in Chinese hamster ovary cells [51]. However, our results strongly indicate that these genes should not be used in different culture systems and at different temperatures in human chondrocytes since their expression is significantly altered by culture temperature and systems. Similar cautions should also be taken in hypoxic conditions [25].

### Effects of culture temperature and system on chondrogenesis related gene expression

The chondrogenic markers, *COL2A1* and *ACAN*, were higher at 37°C than at 32°C and 41°C in both culture systems and more prominent in the pellet culture system (Fig 4A and 4C). These results indicate that the culture temperature of 37°C may promote chondrogenesis better than 32°C and 41°C although the de-differentiation marker, *COL1A1*, was also slightly higher at 37°C in the monolayer culture system (Fig 4B). *SOX9* is a transcription factor that plays a key role in chondrogenesis and skeletogenesis [52,53]. *SOX9* has been shown to directly regulate the expression of *COL2A1* and *ACAN* through enhancing their promoter/enhancer activity [54,55]. However, in our study, the alteration of *COL2A1* and *ACAN* expression by culture temperatures did not coincide with that of *SOX9* (Fig 4A, 4C, and 4D). It has been reported that chondrogenesis is mediated by *SOX9*-independent pathways [56]. These *SOX9*-independent pathways might also be involved in this study. In comparison with the monolayer culture system, *COL2A1* and *SOX9* were dramatically enhanced in the pellet culture system (Fig 4A and 4D). These results are consistent with those of previous reports [57,58]. However, in the pellet culture system, *ACAN* was notably suppressed compared to the monolayer culture system (Fig 4C). These results are inconsistent with those of previous reports [8,9,25,58,59]. However, some studies showed that, when chondrocytes are cultured in the pellet culture system in hypoxic conditions, *ACAN* expression was enhanced compared to that in the monolayer culture system. However, when chondrocytes were cultured in the pellet culture system in normoxic conditions, *ACAN* expression was repressed compared to that in the monolayer culture system [60–62]. Thus, the difference in oxygen tension might be related to *ACAN* repression in this study. Another possibility is that *ACAN* would be regulated by surrounding aggrecan deposition as a negative feedback. Higher aggrecan deposition in the pellet might negatively regulated *ACAN*. To elucidate this possibility, further investigations are needed.

We previously investigated the effects of culture temperature on the chondrocyte phenotype using immature porcine chondrocytes and revealed that *COL2A1* was significantly enhanced at 41°C when compared to 37°C in monolayer [32] and pellet culture systems [31]. In contrast, this study using mature human chondrocytes showed that *COL2A1* expression was significantly repressed at 41°C in both systems, indicating the existence of differences in response to culture temperatures between immature porcine chondrocytes and mature human chondrocytes.

Our study has few limitations. First, our results assessing the metabolic changes and chondrogenic phenotype were obtained by measuring mRNA levels, not protein or activity levels. Since mRNA expression level does not always correlate with protein synthesis level [59], we should also confirm the effects of culture temperature on protein levels. Secondly, detailed signaling cascades involved in the effect of culture temperature and system on chondrocyte metabolism and phenotype remain unclear, although our results indicate that *SOX9*-independent pathway might be involved. Finally, we only analyzed cells obtained from one individual. Therefore, in order to generalize our findings, larger studies are warranted in the future.

## Conclusions

We elucidated the effects of different culture temperatures on human chondrocyte metabolism and phenotype in 2D and 3D culture systems by assessing mRNA expression. Our results indicate that chondrocyte metabolism, estimated by the amount of total RNA and cellular respiration-related gene expression, was maintained or enhanced at 32°C compared to that at 37°C in both monolayer and pellet culture systems. On the other hand, the expression of chondrogenic phenotype-related genes was higher at 37°C compared to that at 32°C. Furthermore, an adverse effect was observed at 41°C. Therefore, manipulating the temperature may be an advantageous approach for regulating chondrocyte metabolic activity and chondrogenesis in human chondrocytes.
